# Preparative Purification of Total Flavonoids from *Sophora tonkinensis* Gagnep. by Macroporous Resin Column Chromatography and Comparative Analysis of Flavonoid Profiles by HPLC-PAD

**DOI:** 10.3390/molecules24173200

**Published:** 2019-09-03

**Authors:** Mengyang Hou, Wenzhong Hu, Zhilong Xiu, Aili Jiang, Lei Men, Kexin Hao, Xingsheng Sun, Duo Cao

**Affiliations:** 1 School of Bioengineering, Dalian University of Technology, Dalian 116024, China; 2 Key Laboratory of Biotechnology and Bioresources Utilization, Ministry of Education, Dalian 116600, China; 3 College of Life Science, Dalian Minzu University, Dalian 116600, China; 4 College of Life Sciences, Yan’an University, Yan’an 716000, China

**Keywords:** *Sophora tonkinensis* Gagnep., purification, quantitation, flavonoids, macroporous resin, column chromatography, HPLC-PAD

## Abstract

For the full development and utilization of *Sophora tonkinensis* Gagnep., this study was primarily intended to established a simple and efficient approach for the preparative purification of total flavonoids from *S. tonkinensis* by macroporous resin column chromatography (MRCC). The adsorption and desorption characteristics of the total flavonoids on ten macroporous resins were first studied, and AB-8 resin was chosen as the most suitable, and the adsorption data were best fitted to the pseudo-second-order kinetics model and Langmuir isotherm model. Furthermore, the technological parameters for the purification of the total flavonoids were optimized using column chromatography. After a sample one-step purification procedure, the content of the total flavonoids increased by about 4.76-fold from 12.14% to 57.82%, with a recovery yield of 84.93%. In addition, the comparative analysis of the flavonoid extracts before and after purification was performed by high-performance liquid chromatography coupled with photodiode-array detection (HPLC-PAD). The results showed that the contents of six major flavonoids in the purified product were all higher than before the purification. Therefore, the AB-8 MRCC established in this work was a promising method for the industrial-scale purification of the total flavonoids from *S. tonkinensis*.

## 1. Introduction

*Sophora tonkinensis* Gagnep., belonging to the genus *Sophora* (Leguminosae), is widely distributed in China, Korea, Japan, and Vietnam. In China, *S. tonkinensis* has been used as folk medicine to treat sore throat, acute pharyngolaryngeal infection, asthma, jaundice, and allergic dermatitis for hundreds of years [1,2]. Phytochemical investigations have shown that *S. tonkinensis* is rich in flavonoids [3,4,5,6,7,8], quinolizidine alkaloids [9,10,11], and arylbenzofurans [12,13]. Recent pharmacological studies have indicated that the flavonoids from *S. tonkinensis* showed pretty good anti-inflammatory [14,15,16], neuroprotective [17], hypoglycemic [18], anti-tumor [8,10], anti-allergic [12], and antioxidant [13] activities. Unfortunately, the flavonoid extracts generally contain many kinds of impurities, such as terpenoids, polysaccharides, proteins, lignans, and so on, which seriously restrict the practical application of flavonoids. Hence, it is essential to develop an effective approach to produce flavonoids with a high purity from *S. tonkinensis*.

There is no doubt that the purification of active components is a significant step for the research and exploitation of natural products, and the biological activity is highly dependent on the chemical components. Ding et al. developed an HPLC fingerprint method for the analysis of the flavonoids in *S. tonkinensis* [19], He et al. developed an high-performance liquid chromatography coupled to photodiode array detector and mass spectrometer (HPLC-DAD-ESI-MS) method for the characterization and determination of the flavonoids in *S. tonkinensis* [20]. In addition, despite the fact that many approaches for the purification of flavonoids from herbs have been developed, such as liquid–liquid extraction [21], silica-gel column chromatography [22], high-speed counter-current chromatography [23], supercritical fluid extraction [24], and preparative-HPLC [25], there is not a satisfactory one, because of the shortcomings in the practical application, such as having a large consumption of organic solvents, complex operations, and poor stability. By contrast, macroporous resin column chromatography (MRCC) is an efficient, low-cost, eco-friendly, and fully-developed technology that is able to meet the needs of industrial production and has been widely applied to enrich and purify the natural active substances, especially flavonoids. For instance, MRCC has been successfully applied to purify flavonoids from *Scorzonera austriaca* [26], *Abelmoschus manihot* flower [27], and *Platycladus orientalis* (L.) Franco [28]. The MRCC has been used for the purification of matrine from *S. tonkinesis* [29]; however, to our knowledge, the research on the purification of flavonoids from *S. tonkinensis* by MRCC has not been discovered in literature.

Consequently, the objective of this work is to explore a simple and efficient technology to purify the total flavonoids from *S. tonkinensis*. Firstly, the screening of optimum resin based on the adsorption and desorption performances was carried out. Then, the adsorption kinetics and isotherms were analyzed so as to explore the potential adsorption mechanism. Sequentially, the optimization of the related process parameters of column chromatography for the purification of the total flavonoids from *S. tonkinensis* was performed. In addition, the change in the contents of the major bioactive flavonoids in the extracts before and after purification was evaluated by high-performance liquid chromatography coupled with photodiode-array detection (HPLC-PAD). This study should contribute to improving the utilization of *S. tonkinensis*.

## 2. Results and Discussion

### 2.1. Resin Selection

**Figure 1:**
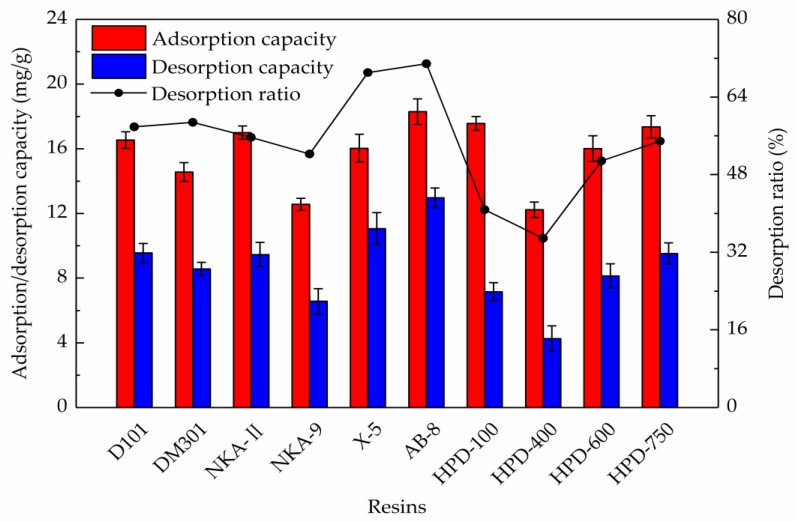
Static adsorption/desorption capacity and desorption ratio of the total flavonoids from *S. tonkinensis* on different resins.

The resins could reversibly adsorb the external organic matters, and the major driving forces were hydrogen bonds and van der Waals forces, and the adsorption properties are exceptionally relevant to their surface area, pore diameter, and polarity [30]. As shown in Figure 1, the adsorption and desorption capacities of the different resins were markedly different. Through comparative analysis, it was found that the adsorption capacities of the AB-8, HPD-100, and HPD-750 resins towards the total flavonoids were 18.30, 17.56, and 17.36 mg/g, respectively, which were comparatively higher than other resins. Overall, the less polar resins showed better adsorption capacities. Furthermore, the desorption capacities of AB-8 and X-5 resins towards the total flavonoids were 12.97 and 11.07 mg/g, respectively. The non-polar X-5 resin had the largest average pore diameter, which was very useful for desorption, but there might be a stronger affinity between the resin and adsorbate molecules, so the desorption capacity of the X-5 resin towards the total flavonoids was lower than that of the AB-8 resin. As a result of the higher adsorption capacity and lower desorption capacity, the HPD-100 resin showed a comparatively lower desorption ratio (40.77%). Conversely, the higher desorption capacities of the AB-8 and X-5 resins led to higher desorption ratios, which were 70.87% and 69.03%, respectively.

In a sum, the AB-8 resin was identified as the best resin for the purification of the total flavonoids from *S. tonkinensis*, because of the best adsorption and desorption properties. Therefore, the AB-8 resin was used for further studies.

### 2.2. Effect of Sample pH on Adsorption Capacity

**Figure 2:**
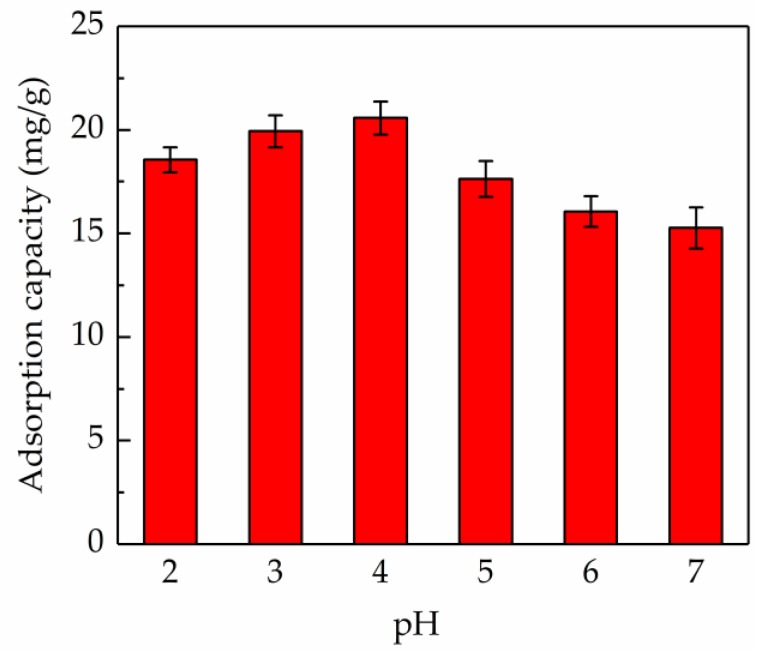
Effect of the sample pH on the adsorption capacity of AB-8 resin.

Assuredly, the adsorption capacity of the adsorbent greatly depends on the solution pH, which has a serious impact on the surface charge characteristics of the adsorbent and the degree of ionization of adsorbate [31]. As shown in Figure 2, it was concluded that the sample pH had a remarkable effect on the adsorption capacity. In the lower range of pH (2.0–4.0), the AB-8 resin presented a better adsorption capacity when compared to a higher pH range (5.0–7.0). This phenomenon might be because the hydrogen bonding interaction between the AB-8 resin and flavonoids molecules was weakened at a higher pH value. When the pH value was 4.0, the adsorption capacity of the AB-8 resin was maximum (20.57 mg/g). Hence, the pH value of the sample solution was regulated to 4.0 in the follow-up experiments.

### 2.3. Adsorption Isotherms

**Figure 3:**
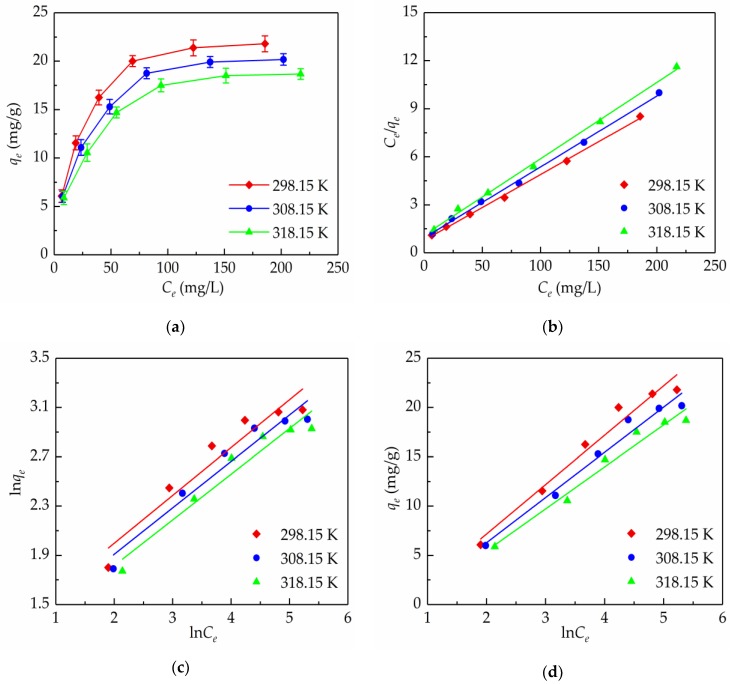
Adsorption isotherms (**a**) and linear correlations on the basis of the Langmuir (**b**), Freundlich (**c**), and Temkin (**d**) models for the total flavonoids from *S. tonkinensis* on the AB-8 resin at 298.15, 308.15, and 318.15 K.

To describe the adsorption performances of the total flavonoids from *S. tonkinensis* on the AB-8 resin, this work studied the relationship between the equilibrium adsorption capacity and the initial concentration of the total flavonoids at 298.15, 308.15, and 318.15 K. As shown in Figure 3a, the adsorption capacity improved rapidly when the concentration of the total flavonoids was lower, and a plateau was reached when the initial concentration of the total flavonoids increased to 0.27 mg/mL, which might be because there were plenty of binding sites on the AB-8 resin so as to adsorb the flavonoids at a lower concentration, and the adsorption sites decreased gradually with increasing the total flavonoid concentration [32]. Thus, the sample solution containing 0.27 mg/mL total flavonoids was used for the subsequent column chromatography experiments. Furthermore, the slope of the *C_e_*–*q_e_* plots decreased with the increasing adsorption temperature, which indicated that the increase in the adsorption temperature was favorable to the adsorption of the total flavonoids onto the AB-8 resin.

Furthermore, to reveal the interaction between the AB-8 resin and flavonoids molecules, the Langmuir, Freudlich, and Temkin isotherm models were usually used for fitting the experimental data in this work. For the Langmuir model, which is based on the basic assumption that all binding sites are energetically equivalent and homogeneously distributed on the adsorption surface, there is no interaction between the adjacent adsorbed molecules and the adsorption in monolayer type. Whereas, the Freudlich model is an empirical equation for describing the adsorption behavior on a heterogeneous surface, and the adsorption in the monolayer, as well as the multi-layer type. Another empirical equation, the Temkin model, takes the interaction between the non-adjacent molecules into account, and assumes that the heat of the adsorption has a linear decreasing trend with an increased degree of surface coverage for the adsorbate on the adsorbent [33].

Plotting *C_e_*/*q_e_* versus *C_e_* (Figure 3b), ln*q_e_* versus ln*C_e_* (Figure 3c), and *q*_e_ versus ln*C_e_* (Figure 3d) would give linear regression lines, respectively, and the parameters of the isotherm models for the adsorption of the total flavonoids on the AB-8 resin are listed in Table 1. *K_L_* represents a tendency, where the adsorbate is attached to an adsorbent, and the larger the *K_L_* value the higher the adsorption energy, and in this study, the values of *K_L_* were decreased with the temperature increasing, which indicated that raising the temperature was adverse to the adsorption. *K_F_* for the Freudlich model is related to the adhesion ability. The values of *K_F_* decreased from 3.3953 to 2.9300 mg/g(L/mg)^1/*n*^ when the adsorption temperature increased from 298.15 to 318.15 K. This indicated that the increase in temperature would decrease the adhesion ability of the total flavonoids onto the AB-8 resin. An exponent, *n*, is a heterogeneity factor, and is also an indicator of the non-linearity degree of adsorption isotherms. As shown in Table 1, all of the values of *n* were between 2 and 10, which indicated that the adsorption was a physical process [34]. Likewise, the Temkin model was also employed to describe the experimental data, and *K_T_* and *B_T_* represent the binding energy and adsorption heat, respectively. The values of *K_T_* decreased from 0.5656 to 0.4865 as the temperature increased from 298.15 to 318.15 K, which indicated that the higher temperature could weaken the binding capability. In addition, compared to the Freudlich and Temkin models, the Langmuir model fitted the experimental data best because of the highest values of the regression coefficient *R*^2^ (0.9977–0.9986) within the range of the temperature tested. This revealed that a monolayer adsorption behavior of the total flavonoids from *S. tonkinensis* happened to the AB-8 resin.

### 2.4. Adsorption Kinetics

**Figure 4:**
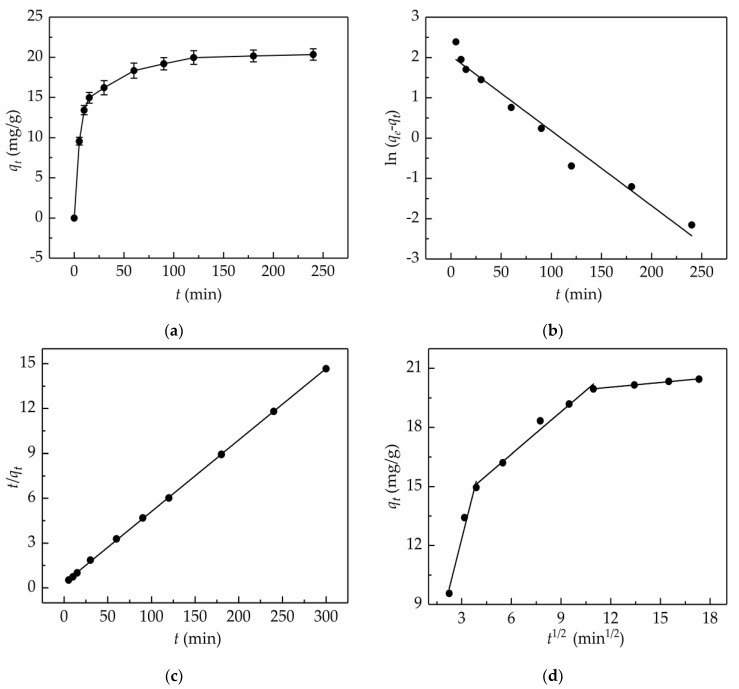
Adsorption kinetic curve (**a**) and linear correlations on the basis of the pseudo-first-order (**b**), pseudo-second-order (**c**), and intra-particle diffusion (**d**) models for the total flavonoids from *S. tonkinensis* on the AB-8 resin at 298.15 K.

In fact, adsorption is a mass transfer process where the adsorbate accumulates on the surface of the adsorbent. The adsorption behavior of a macroporous resin depends on its geometry and material properties. The electrostatic interaction, van der Waals force, and hydrogen bond were the main interaction forces contributing to the binding between the adsorbate and adsorbent. The adsorption process was fairly complicated; hence, to better comprehend the adsorption mechanism, such as the relationship between the adsorption capacity and the time of the adsorption on the AB-8 resin, and the possible rate-determining step of adsorption, adsorption kinetics was researched. As illustrated in Figure 4a, within the first 15 min of the adsorption process, the adsorption capacity increased expeditiously; later increased moderately; and finally, the equilibrium was reached after about 120 min.

The pseudo-first-order model holds that the adsorption rate is proportional to the number of unoccupied sites involving no the interaction between the adsorbents molecules, and the maximum adsorption capacity depends on the saturated monolayer of adsorbate on the adsorbent surface. Whereas the pseudo-second-order model states that the adsorption rate depends on the ratio of the occupied adsorption sites to the unoccupied adsorption sites, and was also related to the interaction between the adsorbent molecules. The most popular formula used for determining the rate-controlling step in a solid/liquid adsorption system is the Weber–Morris intra-particle diffusion model, which assumes that the overall speed of adsorption is related to the physical force or chemical bond between the adsorbate and adsorbent [35].

By fitting the experimental data with kinetic models (i.e., analyzing the plots of ln(*q*_e_-*q_t_*) versus t (Figure 4b), *t*/*q_t_* versus *t* (Figure 4c), and *q_t_* versus *t*^1/2^ (Figure 4d)), the kinetic equations and relevant parameters for the adsorption of the total flavonoids from the *S. tonkinensis* on the AB-8 resin were calculated and are listed in Table 2. It was found that the theoretical maximum adsorption capacity (20.92 mg/g) calculated from the pseudo-second-order model was fairly close to the experimental value (20.46 mg/g), whereas the theoretical maximum adsorption capacity (7.70 mg/g) calculated from the pseudo-first-order model was much less than the experimental value. Moreover, the pseudo-second-order model yielded relatively higher *R*^2^ values (0.9999) than the pseudo-first-order model (0.9696). Taken together, this adsorption process was in well agreement with the pseudo-second-order model. In the case of the intra-particle diffusion model, three consecutive steps were involved, more specifically, a sharper line segment represented the diffusion of the total flavonoids through the solution to the external surface of the AB-8 resin, or the boundary layer diffusion of the flavonoids. Then, a line segment that sloped slightly represented the movement of the flavonoids into the interior part of the AB-8 resin. The final equilibrium stage was the adsorption of flavonoids onto the interior surface of the AB-8 resin. It is worth mentioning that the value of *I*, the *y*-intercept, can indicate the thickness of the boundary layer; the larger the y-intercept, the smaller the contribution of the intra-particle diffusion. When *I* = 0, there was no boundary layer thickness, and the intra-particle diffusion model is the only rate-limiting step [36]. As seen from Figure 4d, the plots did not pass through the origin, which implied that the rate-limiting step was not only the intra-particle diffusion, but also the adsorption or boundary layer diffusion involved in the adsorption process.

### 2.5. Column Chromatography

#### 2.5.1. Dynamic Breakthrough Curves

**Figure 5:**
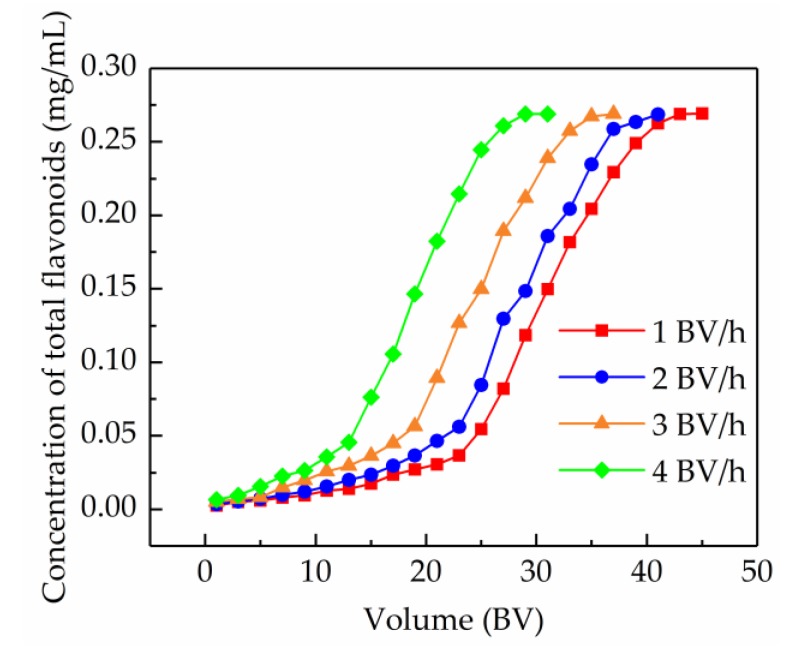
Dynamic breakthrough curves of the total flavonoids from *S. tonkinensis* on the column packed with the AB-8 resin at different flow rates.

The breakthrough curve can give significant evidence for the further analysis of the dynamic characteristics of an adsorption fixed-bed column, and has important implications for the optimizing and designing of a purification process. In this study, the breakthrough point was defined as the time when the concentration of the total flavonoids in the effluent achieved 10% of the initial concentration [37]. The breakthrough curves on the AB-8 resin-packed column at different flow rates are shown in Figure 5. It was found that the lower flow rate of the sample solution, the better the adsorption performance of the AB-8 resin. In other words, the breakthrough point delayed with the decrease of the flow rate, which was probably because the prolonged contact time was conducive to mass transfer. It was also found that the difference in the appearance of the breakthrough points obtained at 1 BV/h and 2 BV/h was subtle. Thus, with a view to enhance the operational efficiency, 2 BV/h was considered as the optimal feed flow rate, and the corresponding breakthrough volume was 16 BV.

#### 2.5.2. Effect of Ethanol Concentration on Desorption Ratio

**Figure 6:**
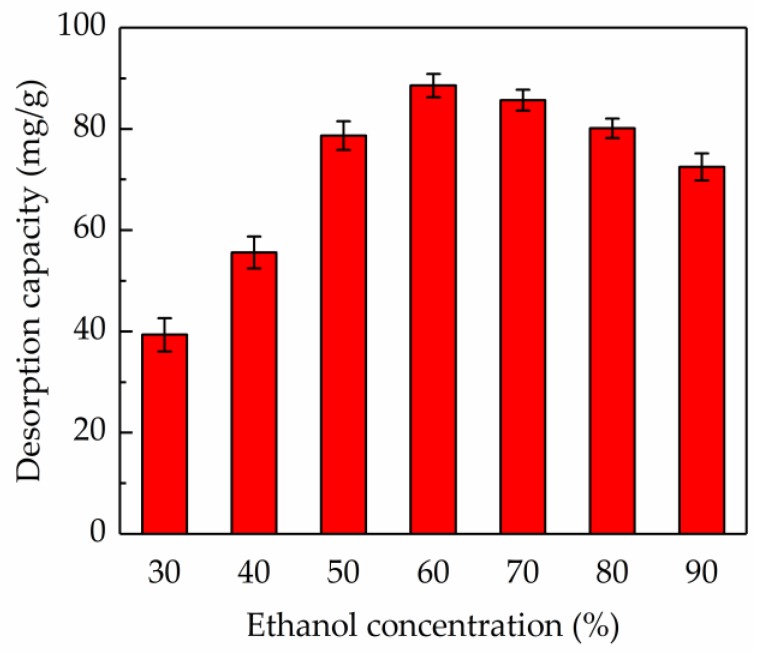
Effect of the ethanol concentration on the desorption capacity of the AB-8 resin.

Figure 6 shows that the ethanol concentration was of great importance to the desorption rate. The desorption ratio of the AB-8 resin for the total flavonoids was increased in the ethanol concentration range of 30–60% (*v*/*v*), and when the ethanol concentration was within 60–90% (*v*/*v*), the desorption ratio showed a decreasing tendency. The highest desorption ratio (88.56%) was obtained when the ethanol concentration was 60% (*v*/*v*). Hence, 60% (*v*/*v*) ethanol was considered as the best eluent.

#### 2.5.3. Dynamic Desorption Curves

**Figure 7:**
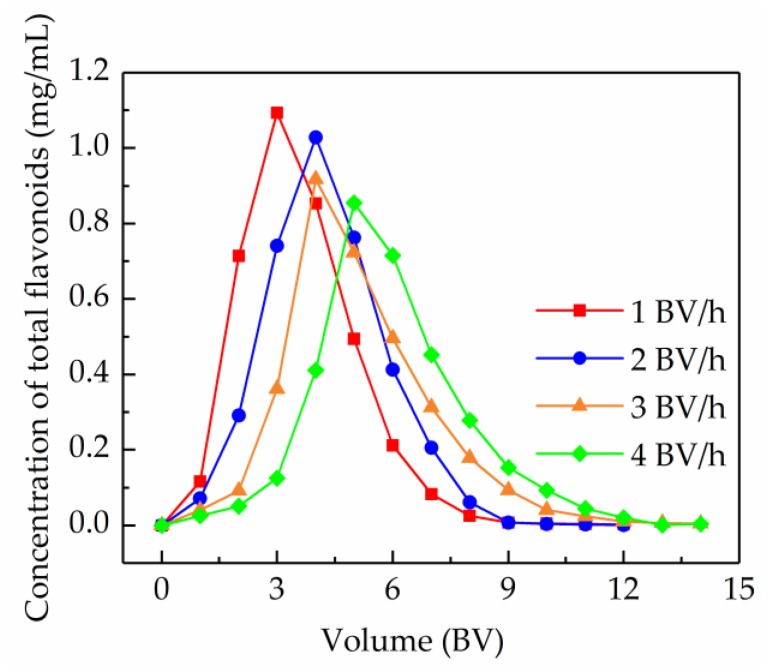
Dynamic desorption curves of the total flavonoids from *S. tonkinensis* on the column packed with the AB-8 resin at different flow rates.

To reduce the eluent consumption and improve the desorption efficiency, research about the dynamic desorption curve is very necessary. In this research, the dynamic desorption curves were obtained by an isocratic elution model at 60% (*v/v*) ethanol, using a flow rate of 1, 2, 3, and 4 BV/h. As shown in Figure 7, a lower elution rate provided more effective desorption. However, the effect on the desorption capacity of the AB-8 resin at 1 and 2 BV/h was almost the same, and the eluent consumption at 1 and 2 BV/h was almost identical when the desorption equilibrium occurred. Therefore, in order to enhance operational efficiency, 2 BV/h was considered as the optimal elution rate, and the corresponding volume of the desorption solution was 9 BV when the total flavonoids was thoroughly desorbed.

### 2.6. Preparative Purification of Total Flavonoids Under Optimized Conditions

The efficiency of the AB-8 resin column chromatography was evaluated in a lab-scale apparatus (1.6 cm ID × 40 cm length) with a bed volume (BV) of 20 mL. The preparative purification procedure was performed according to the above optimization conditions. First, 16 BV of a sample solution (pH 4.0) containing 0.27 mg/mL of the total flavonoids was fed into the AB-8 resin column at a flow rate of 2 BV/h. Then, 10 BV of deionized water was used to wash the column. Then, 9 BV of 60% aqueous ethanol was used for the desorption of flavonoids, and the flow rate was 2 BV/h. The eluate was collected and concentrated, a product consisting 57.82% total flavonoids was obtained, and a recovery of 84.93% was reached, which validated the feasibility and reliability of the AB-8 resin column chromatography established in this work.

### 2.7. Comparative Analysis of Flavonoid Profiles by HPLC-PAD

**Figure 8:**
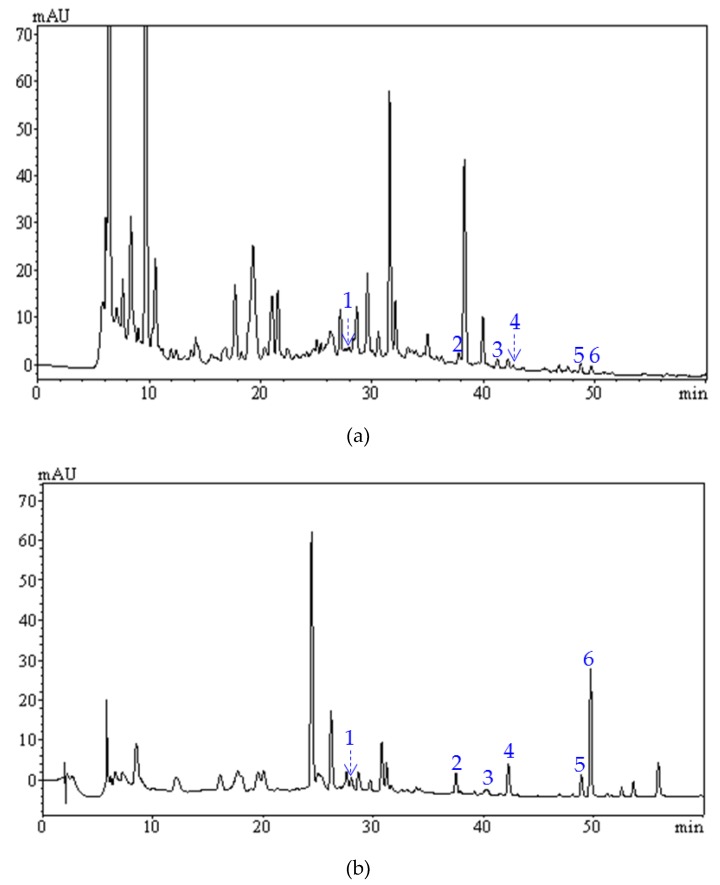
High-performance liquid chromatography coupled with photodiode-array detection (HPLC-PAD) chromatograms of the flavonoid extracts from *S. tonkinensis* before (**a**) and after (**b**) purification at 254 nm. Peaks 1, 2, 3, 4, 5, and 6 represent rutin, trifolirhizin, quercitrin, quercetin, maackiain, and formononetin, respectively.

Flavonoids from *S. tonkinensis*, including maackiain, trifolirhizin, quercetin, formononetin, quercitrin, and rutin, have lots of important biological activities, and the qualitative and quantitative analysis of these flavonoids in *S. tonkinensis* extracts will be of great significance for quality evaluation and bioactivity study. As shown in Figure 8, there was a great difference in the HPLC-PAD chromatograms of *S. tonkinensis* extracts before and after purification. A total of six flavonoids (maackiain, trifolirhizin, quercetin, formononetin, quercitrin, and rutin) were identified through a comparative analysis of the retention time and ultraviolet absorption spectrum of the chromatographic peak, and those of the reference substance. Furthermore, six flavonoids in the extracts before and after purification were quantified simultaneously through the external standards method, and the contents of six active constituents in the extracts before and after purification are summarized in Table 3. It was found that the contents of each flavonoid increased through a one-step purification procedure; in particular, the content of formononetin increased by 12.56-fold. The increase in the contents of these active ingredients should be helpful for further separation and bioactivity study. In addition, this study confirmed the previous report that trifolirhizin, quercetin, maackiain, and formononetin were the main flavonoids present in *S. tonkinensis* [20].

## 3. Materials and Methods

### 3.1. Chemicals and Reagents

Ten resins, including D101, DM301, HPD-100, NKA-II, NKA-9, AB-8, X-5, HPD-400, HPD-600, and HPD-750 were purchased from Cangzhou Bonchem Co., Ltd. (Hebei, China), and their physical parameters are listed in Table 4.

The rutin, maackiain, trifolirhizin, quercetin, formononetin, and quercitrin standards were purchased from Shanghai Yuanye Biotechnology Co., Ltd. (Shanghai, China). The HPLC-grade methanol and trifluoroacetic acid (TFA) were obtained from Sigma-Aldrich Co. Ltd. (St. Louis, MO, USA). All of the other analytical-grade chemicals were purchased from Tianjin Kemio Chemical Co. (Tianjin, China).

### 3.2. Preparation of Sample Solutions

*Sophora tonkinensis* Gagnep. was collected from Guangxi Province, China, and the collected sample was identified by Associate Professor Xiaozhong Chen (Heilongjiang University of Chinese Medicine, Harbin, China). Dried *S. tonkinensis* was powdered and extracted thrice with 70% (*v/v*) ethanol under reflux, each time for 2 h. Subsequently, the extracts were filtered and concentrated under reduced pressure at 50 °C in order to yield a residue. The residue containing 12.14% of the total flavonoids was then suspended in water so as to obtain sample solutions with different concentrations of total flavonoids (0.07–0.40 mg/mL).

### 3.3. Determination of Total Flavonoids Content

The method used to measure the content of the total flavonoids was based on the method previously reported, with slight modifications [38]. In brief, 1 mL of the sample solution was placed in a 5 mL volumetric flask, then 150 μL of 5% (*w/v*) NaNO_2_ was added and mixed. Six minutes later, 150 μL of 10% (*w*/*v*) Al(NO_3_)_3_ was added, and the mixture was mixed for 6 min, followed by the addition of 1 mL of 4% (*w*/*v*) NaOH. Afterwards, the solution was supplemented to 5 mL with 30% (*v*/*v*) aqueous ethanol, and then rested for 15 min. The absorbance value was detected at 510 nm using a UV-2600 spectrophotometer (Shimadzu, Kyoto, Japan). The calibration curve was obtained based on the absorbance values of a series of rutin standard solutions, which showed a good linearity (A=11.1353C+0.0078, *R*^2^ = 0.9998) in the range of 18.12–63.42 μg/mL, where *A* was the absorbance value and *C* was the total flavonoids content (mg/mL).

### 3.4. Comparison of Adsorption Capacity, Desorption Capacity and Ratio

In order to select an optimal resin to purify the total flavonoids from the *S. tonkinensis*, the static adsorption and desorption tests were carried out. Briefly, 1 g of each resin was placed into 250 mL conical flasks, then, 100 mL of the sample solution (total flavonoids concentration 0.27 mg/mL) was added separately. The conical flasks were shaken (150 rpm) using a shaking table at 298.15 K for 12 h. After the adsorption equilibrium had been reached, the sample solutions were moved out of the conical flasks and the resins were rinsed with distilled water thoroughly. Then, 100 mL of 95% (*v/v*) ethanol was added for the desorption, and the conical flasks were shaken for 12 h under the same conditions.

The influence of the sample solution pH on the adsorption capacity of the selected resin was researched, as follows: 1 g of the selected resin was blended with 100 mL of the sample solution, with the pH adjusted to 2.0–7.0, by the addition of diluted HCl or NaOH, and then the adsorption capacity was measured under the same conditions.

The equilibrium adsorption capacity was worked out according to the following equation:

(1) $$q_{e}=\frac{(C_{0}-C_{e})V_{i}}{m}$$

where *q_e_* (mg/g) is the equilibrium adsorption capacity, *C_0_* (mg/mL) is the initial concentration of total flavonoids, *C_e_* (mg/mL) is the equilibrium concentration of the total flavonoids, *V_i_* is the initial volume of sample solution, and *m* (g) is the dry weight of resin.

The equilibrium desorption capacity and the desorption ratio were calculated by the following equations:

(2) $$q_{d}=\frac{C_{d}V_{d}}{m}$$

(3) $$D\;(\%)=\frac{C_{d}V_{d}}{(C_{0}-C_{e})V_{i}}\times 100$$

where *q_d_* (mg/g) represents the desorption capacity, *C_d_* (mg/mL) represents the concentration of the total flavonoids in the desorption solution, *D* (%) represents the desorption ratio, and *V_d_* represents the volume of the desorption solution.

### 3.5. Adsorption Isotherms

The adsorption isotherms of total flavonoids from *S. tonkinensis* on AB-8 resin were studied by blending 1 g of resin with 100 mL of sample solutions with different total flavonoids concentrations (0.07–0.40 mg/mL), and the mixtures were shaken continuously at a speed of 150 rpm for 12 h, at 298.15, 308.15, and 318.15 K, respectively.

For a better understanding of the adsorption behaviors, the equilibrium adsorption data were analyzed using the Langmuir, Freundlich, and Temkin isotherms models.

Langmuir equation:

(4) $$\frac{C_{e}}{q_{e}}=\frac{1}{K_{L}q_{m}}+\frac{C_{e}}{q_{m}}$$

Freundlich equation:

(5) $$\ln q_{e}=\frac{1}{n}\ln C_{e}+\ln K_{F}$$

Temkin equation:

(6) $$q_{e}=B_{T}\ln K_{T}+B_{T}\ln C_{e}$$

where *q_m_* (mg/g) represents the theoretical maximum adsorption capacity, *K_L_* (L/mg) is the Langmuir adsorption constant, *n* and *K_F_* [mg/g(L/mg)^1/*n*^] are the Freundlich constants, and *K_T_* (L/mg) and *B_T_* (J/mol) are Temkin constants.

### 3.6. Adsorption Kinetics

The adsorption kinetics of the total flavonoids from *S. tonkinensis* on the AB-8 resin were studied by blending 1 g of resin with 100 mL of the sample solution (total flavonoids concentration 0.27 mg/mL) with a shaking speed of 150 rpm for 4 h at 298.15 K. At different adsorption time intervals (0, 5, 10, 15, 30, 60, 90, 120, 180, and 240 min), the adsorption capacity of the AB-8 resin was measured.

For a better understanding of the mass-transfer mechanism, the pseudo-first-order kinetic model, pseudo-second-order kinetic model, and Weber–Morris intra-particle diffusion model were applied to analyze the adsorption process, and the equations used were as follows:

The pseudo-first-order kinetic model:

(7) $$\frac{dq_{t}}{dt}=k_{1}(q_{e}-q_{t})$$

Equation (7) can be integrated as follows:

(8) $$\ln(q_{e}-q_{t})=-k_{1}t+\ln q_{e}$$

The pseudo-first-order kinetic model:

(9) $$\frac{dq_{t}}{dt}=k_{2}{\left(q_{e}-q_{t}\right)}^{2}$$

Equation (9) can be integrated as follows:

(10) $$\frac{t}{q_{t}}=\frac{1}{k_{2}{q_{e}}^{2}}+\frac{t}{q_{e}}$$

The Weber–Morris intra-particle diffusion model:

(11) $$q_{t}=k_{i}t^{1/2}+I$$

where *k*_1_ and *k*_2_ represent the rate constants of the pseudo-first-order kinetic model and pseudo-first-order kinetic model, respectively. *k_i_* and *I* are the constants of the Weber–Morris intra-particle diffusion model.

### 3.7. Optimization of Resin Column Chromatography Conditions

Dynamic adsorption and desorption tests were performed using lab-scale glass columns (1.6 cm ID × 40 cm length), which were packed with the AB-8 resin, and the BV was 20 mL. In order to study the dynamic breakthrough curves, the sample solutions were loaded onto the column at different flow rates (1, 2, 3, and 4 BV/h), and the concentration of the total flavonoids in the effluent was detected until the adsorption saturation point was reached. In the process of dynamic desorption, the optimal desorption solution was chosen based on the following method: the columns loaded with the sample were first washed using 10 BV of deionized water, and then eluted with 10 BV of 30–90% (*v/v*) ethanol solutions, respectively, then the desorption capacities of the ethanol solutions at different concentrations were compared. Subsequently, to determine the optimal volume and flow rate of the desorption solution, the columns loaded with the sample were eluted with an optima desorption solution at different flow rates (1, 2, 3, and 4 BV/h), respectively, and the total flavonoid concentrations in the desorption solutions were analyzed comparatively.

### 3.8. HPLC-PAD Analysis of S. tonkinensis Extract Before and After Purification

The chemical profiles of the flavonoid extracts from *S. tonkinensis* before and after purification were analyzed using a LC-20AD (Shimadzu, Tokyo, Japan) system equipped with a photodiode array detector (SPD-M20A). A YMC-Pack ODS-A C18 column (4.6 mm × 250 mm, 5 μm) was used for separation at 30 °C. The mobile phase consisted of methanol (A) and 0.05% TFA in water (B) with a gradient elution as follows: 0–40 min, 30–75% B; 40–60 min, 75–80% B. The total flow rate was 0.5 mL/min, and the detection wavelength was set at 284 nm.

## 4. Conclusions

Among the researched resins, the AB-8 resin afforded the most excellent absorption property for the total flavonoids from the *S. tonkinensis*. The kinetics study revealed that the adsorption process best fitted the pseudo-second-order model at 298.15 K. The adsorption isotherm was delineated best by the Langmuir isotherm model. In addition, a simple, eco-friendly, and efficient method for the purification of the total flavonoids from *S. tonkinensis* was developed. The content of the total flavonoids was increased 4.76-fold, with an effective recovery of 84.93%, through one-step AB-8 column chromatography. In addition, the HPLC-PAD analysis indicated that the contents of the six flavonoids (rutin, maackiain, trifolirhizin, quercetin, formononetin, and quercitrin) in the *S. tonkinensis* extracts were dramatically improved after purification. Therefore, this study provided a potential approach for the large-scale purification of the total flavonoids from *S. tonkinensis*, and the HPLC-PAD method established could be applied for quality control in the purification process.

## Figures and Tables

**Table 1:** Adsorption isotherm equations and parameters of the total flavonoids from the *S. tonkinensis* on the AB-8 resin.

Model	*T* (K)	Equations	Parameters		
			*K_L_* (L/mg)	*q_m_* (mg/g)	*R* ^2^
Langmuir	298.15	$\frac{C_{e}}{q_{e}}=0.0411C_{e}+0.7806$	0.0527	24.33	0.9986
	308.15	$\frac{C_{e}}{q_{e}}=0.0443C_{e}+0.9388$	0.0472	22.57	0.9981
	318.15	$\frac{C_{e}}{q_{e}}=0.0477C_{e}+1.1071$	0.0431	20.96	0.9977
			***K_F_* [mg/g(L/mg)^1/*n*^]**	**n**	***R*^2^**
Freundlich	298.15	$\ln q_{e}=0.3882\ln C_{e}+1.2224$	3.3953	2.5760	0.9215
	308.15	$\ln q_{e}=0.3767\ln C_{e}+1.1559$	3.1769	2.6546	0.9432
	318.15	$\ln q_{e}=0.3710\ln C_{e}+1.0750$	2.9300	2.6954	0.9439
			***K_T_* (L/mg)**	***B_T_* (J/mol)**	***R*^2^**
Temkin	298.15	$q_{e}=5.0133\ln C_{e}-2.8570$	0.5656	5.0133	0.9696
	308.15	$q_{e}=4.5858\ln C_{e}-2.8858$	0.5330	4.5858	0.9736
	318.15	$q_{e}=4.2617\ln C_{e}-3.0703$	0.4865	4.2617	0.9704

**Table 2:** Thermodynamic parameters for the adsorption of the total flavonoids from *S. tonkinensis* on the AB-8 resin.

Kinetics Model	Regression Equations	Parameters
Pseudo-first-order	$\ln(q_{e}-q_{t})=-0.0186t+2.0411$	*k*_1_ = 0.0186 min^−1^ *Q_e_* = 7.70 mg/g *R*^2^ = 0.9696
Pseudo-second-order	$\frac{t}{q_{t}}=0.0478t+0.3289$	*k*_2_ = 6.9469 × 10^−3^ g/(mg·min) *Q_e_* = 20.92 mg/g *R*^2^= 0.9999
Intra-particle diffusion (first stage)	$q_{t}=3.3432t^{1/2}+2.3129$	*k_i_* = 3.3432 mg/(g·min^1/2^) *I* = 2.3129 mg/g *R*^2^ = 0.9721
(second stage)	$q_{t}=0.7191t^{1/2}+12.3295$	*K_i_* = 0.7191 mg/(g·min^1/2^) *I* = 12.3295 mg/g *R*^2^ = 0.9843
(third stage)	$q_{t}=0.0797t^{1/2}+19.0885$	*K_i_* = 0.0797 mg/(g·min^1/2^) *I* = 19.0885 mg/g *R*^2^ = 0.9966

**Table 3:** Comparison of the flavonoid contents in the extracts from *S. tonkinensis* before and after purification.

Compounds	Retention Time (min)	Content (%)		Recovery
		Before Purification	After Purification	
Rutin	28.362	0.014%	0.036%	83.47%
Trifolirhizin	37.825	0.756%	3.198%	86.51%
Quercitrin	40.641	0.026%	0.032%	84.22%
Quercetin	42.103	0.007%	0.067%	91.53%
Maackiain	48.759	1.617%	7.842%	87.16%
Formononetin	49.537	0.067%	0.842%	85.90%

**Table 4:** Physical properties of the tested macroporous resins.

Resins	Particle Size (mm)	Surface Area (m^2^/g)	Average Pore Diameter (nm)	Polarity
NKA-9	0.3~1.25	170~250	15.5~16.5	Polar
NKA-II	0.3~1.25	160~200	14.5~15.5	Polar
HPD-600	0.3~1.25	550~600	8.0~9.0	Polar
DM301	0.3~1.25	330~380	9.0~11.0	Middle polar
HPD-400	0.3~1.25	500~550	7.5~8.0	Middle polar
HPD-750	0.3~1.25	650~700	8.5~9.0	Middle polar
AB-8	0.3~1.25	450~530	13.0~14.0	Weak polar
HPD-100	0.3~1.25	650~700	8.5~9.0	Non-polar
D101	0.3~1.25	600~700	10.0~12.0	Non-polar
X-5	0.3~1.25	500~600	21.0~23.0	Non-polar
